# Contrasting identity-by-descent estimators, association studies, and linkage analyses using the Framingham Heart Study data

**DOI:** 10.1186/1753-6561-3-s7-s102

**Published:** 2009-12-15

**Authors:** Elizabeth E Marchani, Yanming Di, Yoonha Choi, Charles Cheung, Ming Su, Frederick Boehm, Elizabeth A Thompson, Ellen M Wijsman

**Affiliations:** 1 Division of Medical Genetics, Department of Medicine, University of Washington, Health Sciences Building, K-253, Box 357720, Seattle, Washington 98195 USA; 2 Department of Statistics, University of Washington, Box 354322, Seattle, Washington 98195 USA; 3 Department of Biostatistics, University of Washington, F-600, Health Sciences Building, Box 357232, 1705 North East Pacific Street, Seattle, Washington 98195 USA

## Abstract

We explored the utility of population- and pedigree-based analyses using the Framingham Heart Study genome-wide 50 k single-nucleotide polymorphism marker data provided for Genetic Analysis Workshop 16. Our aims were: 1) to compare identity-by-descent sharing estimates from variable amounts of data; 2) to apply each of these estimates to a case-control association study designed to control for relatedness among samples; and 3) to contrast these results to those obtained using model-based and model-free linkage analysis methods.

## Background

The study of quantitative traits has led to the development of tools of varying complexity designed to identify chromosomal regions associated with disease. This has been coupled in recent years by the increasing availability of data sets that include hundreds of thousands of markers. We investigated the utility of using more data and more sophisticated analyses by applying analytical methods of variable complexity to a single data set and comparing their results. We also estimated the same statistics using variable amounts of marker data to investigate the influence of the amount of marker data on the estimates. Generally speaking, we asked whether we really benefit from these new tools and large data sets, or do they simply increase the complexity of our research? More specifically, we: 1) compared estimates of identity-by-descent (IBD) sharing from increasing amounts of marker data; 2) evaluated how well different IBD estimates corrected a case-control association study for relatedness; and 3) compared the results of our case-control study and various linkage analyses to contrast any signals of association between genotype and phenotype.

## Methods

### Genetic map and marker data

We focused exclusively on the Genetic Analysis Workshop (GAW) 16 50 k marker data, and primarily analyzed only chromosome 7. We matched the position of each chromosome 7 marker to the sex-averaged Kosambi map sequence position on the Rutgers map [1], and then converted those positions to a Haldane map. Markers within <0.01 cM of each were given unique and sequential map positions to obtain non-overlapping map positions.

We filtered markers with >3% missing data and with minor allele frequency <0.05. We used chi-square tests to test the null hypothesis of Hardy-Weinberg equilibrium, and removed markers yielding the largest 1% of the test statistics, leaving 2132 markers on chromosome 7. A "thinned" marker panel was obtained by selecting approximately every tenth marker from this "dense" filtered marker set, preferentially selecting markers with higher rare allele frequencies among founders because they are more suitable for linkage analysis. The final thinned data set included 214 markers on chromosome 7 (and 3465 genome-wide markers) with a marker density of ~1 per cM.

### Pedigree data cleaning

To ensure compatibility with the linkage analysis programs in the MORGAN package [2], we merged the two members of each of 25 monozygotic twin pairs. Parents missing pedigree information who were referenced by at least two family members were given records of their own. Mendelian-inconsistent genotypes were identified by Loki 2.4.7 [3] and recoded as missing genotypes for all members of each affected pedigree. All individuals sharing a pedigree number could not necessarily be connected, so we split the larger pedigree into smaller pedigrees generated by available parent-offspring relationships.

### Phenotype data refinement

For linkage-based analyses, we focused on high-density lipoprotein level (HDL) and chromosome 7 due to previous evidence of linkage within the Framingham Heart study (FHS) [4-8]. We used observations from Exam 11 for the Original Cohort, and Exam 1 for the Offspring and Generation 3 Cohort, age-matching the second and third generations to maximize the number of individuals in our study. Height was imputed from Exam 7 of the Original Cohort data when it was missing from Exam 11 in order to calculate body mass index (BMI). We fit linear regression models to adjust HDL for age, BMI, sex, cholesterol treatment status, and cohort.

### Quantitative trait locus models

We performed Bayesian oligogenic segregation analysis using the software package Loki 2.4.7 [3] to identify and describe models for quantitative trait loci (QTLs) associated with the adjusted HDL phenotype. The QTL with the largest effect size (A allele frequency = 0.76, AA genotype effect = -1.39, Aa genotype effect = -1.75, aa genotype effect = 24.21, variance due to the QTL = 12.04, additive variance = 13.53, dominance variance = 23.54) was incorporated into the MORGAN [2] lm_multiple analysis described below. We used the posterior distribution of these models to generate a sample of simulated traits for use in empirical significance testing [9].

### IBD sharing and kinship from population and pedigree data

Without reference to the pedigree structures, we estimated *k*-coefficients, where *k*_*i *_is the probability that *i *alleles are shared IBD, using the thinned chromosome 7 and genome-wide panels of markers, as well as subsets of 214 and 1000 genome-wide markers. We estimated *k*-coefficients for all possible pairs of independent people (*n *= 1827), using all founders in the pedigrees and other unrelated individuals, and for all pairs of individuals within each pedigree. Kinship coefficients, Φ, were subsequently computed as Φ = 0.25*k*_1 _+ 0.5*k*_2 _and pairs of individuals with Φ > 0.2 were noted.

We selected four pairs of individuals showing high apparent relatedness as estimated from the thinned chromosome 7 markers while differing with respect to pedigree numbers. For each pair, the dense chromosome 7 markers were used to detect IBD segments using the model of Thompson [10]. We used a prior marginal pairwise IBD probability 0.1, and for an IBD-change rate parameter giving a prior expected length of chromosome in a particular IBD state of 1 cM, averaged over the nine possible IBD states in accordance with their marginal prior probabilities. The dense chromosome 7 data set was also used to flag tracts of homozygous markers (>9 SNPs in a row) shared between each of these four pairs of individuals.

We used a new "case-control" study design that corrects for relatedness (both known and estimated as cryptic kinship) within the sample [11], choosing 838 "cases" and 844 "controls" from the upper and lower 15^th ^percentiles of the trait distribution in the full data set. The correction for relatedness essentially eliminates inflated test statistics resulting from inclusion of related individuals. We corrected the naïve chi-square statistic *p*-values using three types of kinship coefficients: pedigree-based prior, pedigree-based posterior, and population-estimated kinship coefficients. The pedigree-based prior was computed based on pedigree structure alone, while the pedigree-based posterior was based on the gl_auto results (described below) that used both pedigree structure and marker data. The dense chromosome 7 marker panel was used for the case-control study, while the thinned chromosome 7 marker panel was used for estimation of kinship coefficients. The population-estimated kinship coefficient was a maximum-likelihood estimate based on the thinned chromosome 7 marker data.

### Linkage analyses

Two MORGAN [2] programs, lm_multiple and gl_auto, were used for lod score analyses and realization of inheritance indicators conditional on marker data, respectively. Options in both programs now allow the multiple-meiosis sampler to be used with the locus sampler, leading to more accurate Markov-chain Monte Carlo (MCMC) sampling of inheritance indicators on large pedigrees [12]. Additionally, both programs have options to run sequentially over pedigrees, permitting easier processing of output on disjoint pedigrees and allowing for exact computation of lod scores on small (<= 14 meioses) pedigrees in lm_multiple, and independent realizations of inheritance indicators in gl_auto. This allows computationally intensive MCMC approximation to be used only where necessary.

Genetic linkage can be detected with pedigree data using inheritance vector realizations. We used the inheritance vectors obtained from gl_auto for two linkage analysis methods: 1) standard variance-components (VC) analysis using SOLAR, and 2) a novel conditional inheritance vector test using the *w*-score [13], which is the expectation over founder genotypes of a maximized likelihood given those founder genotypes, to test whether we could resolve the number of causal loci in a region of interest indicated by the VC results. The *w*-score analyses were performed only on the size 4-9 pedigrees, while VC analysis was performed on this subset as well as on all pedigrees for comparison. We summarized the results using randomized *p*-values for the conditional test [14] and empirical *p*-values for the VC analysis through trait simulation and the inheritance vectors described above [9]. We also performed three Bayesian oligogenic joint segregation and linkage analyses on all pedigrees using Loki 2.4.7 [3], where every 100^th ^out of 500,000 iterations were used to compute Bayes' factors for the presence of a QTL within each 2-cM bin.

## Results

### IBD sharing and kinship from population and pedigree data

**Figure 1:**
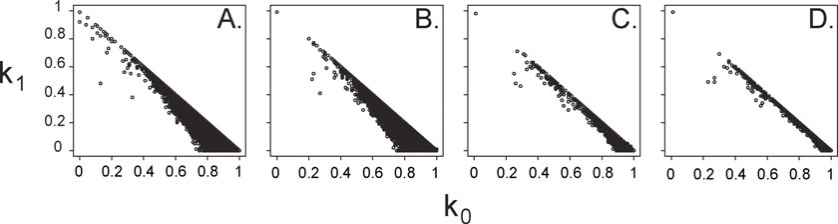
**IBD estimates using variable amounts of marker data**. IBD estimates using 214 markers on chromosome 7 (A) or 214 (B), 1000 (C), or 3465 (D) markers from the whole genome.

We found several pairs of independent individuals that share non-zero average IBD (Figure 1), but the number of such pairs was dependent on the number of markers used. While 26 pairs of independent people had kinship coefficients >0.2 using the 214 markers on chromosome 7, only 4 such pairs were identified using 3465 genome-wide markers. Although many of these 26 pairs include individuals sharing the same pedigree number as defined in the GAW16 data and represent cases where relationship information could not be reconstructed from the pedigree file alone, we also found that several pairs from different pedigrees had non-zero *k*_1 _or *k*_2 _values for chromosome 7. This indicates there is non-negligible relatedness among independent individuals across pedigrees.

Detailed analysis of four such pairs of independent individuals with unique pedigree numbers are summarized in Table 1. Estimates of *k*_1 _decreased as the number of markers used increased for two of the four pairs of individuals. The IBD segments analysis estimated the full nine-state IBD probabilities along the chromosome, with the average of these values reported in Table 1. Two large IBD segments with very sharply defined boundaries were detected for pair 10895 and 9894. With probabilities very close to 1, this pair shows both genes IBD from positions 29.6 cM to 70.0 cM and from 133 cM to 160 cM. The within-individual IBD probabilities are surprisingly high, also indicating broad regions of homozygosity among these individuals on chromosome 7. Results of the segments analysis were robust to the IBD change-rate parameter. This is corroborated by the observation of multiple shared tracts of homozygosity within each pair.

**Table 1:** Estimated proportions of IBD for four putatively unrelated pairs of individuals

	Pairs															
	
	10895 and 9894				13728 and 11898				19185 and 11156				23487 and 25107			
				
	I^b^	S^c^	g^d^	G^e^	I	S	g	G	I	S	g	G	I	S	g	G
k_0_^a^	0.13	0.11	0.27	0.26	0.44	0.39	0.43	0.53	0.45	0.33	1.00	0.98	0.17	0.19	0.39	0.50
k_1_	0.48	0.44	0.41	0.52	0.45	0.52	0.45	0.36	0.51	0.62	0.00	0.02	0.70	0.70	0.61	0.49
IBD - within individuals	-^f^	0.16	-	-	-	0.37	-	-	-	0.26	-	-	-	0.28	-	-
No. homozygous tracts	3	-	-	-	5	-	-	-	3	-	-	-	7	-	-	-
Range of tract length (SNPs)	[10:14]	-	-	-	[6:12]	-	-	-	[5:11]	-	-	-	[3:21]	-	-	-

^a^k_0 _and k_1 _are the probability of sharing 0 and 1 alleles IBD, respectively

^b^I, 214 chromosome 7 markers

^c^S, chromosome 7 segments analysis

^d^g, 214 genome-wide markers

^e^G, 3465 genome-wide markers

^f^-, Not available

**Figure 2:**
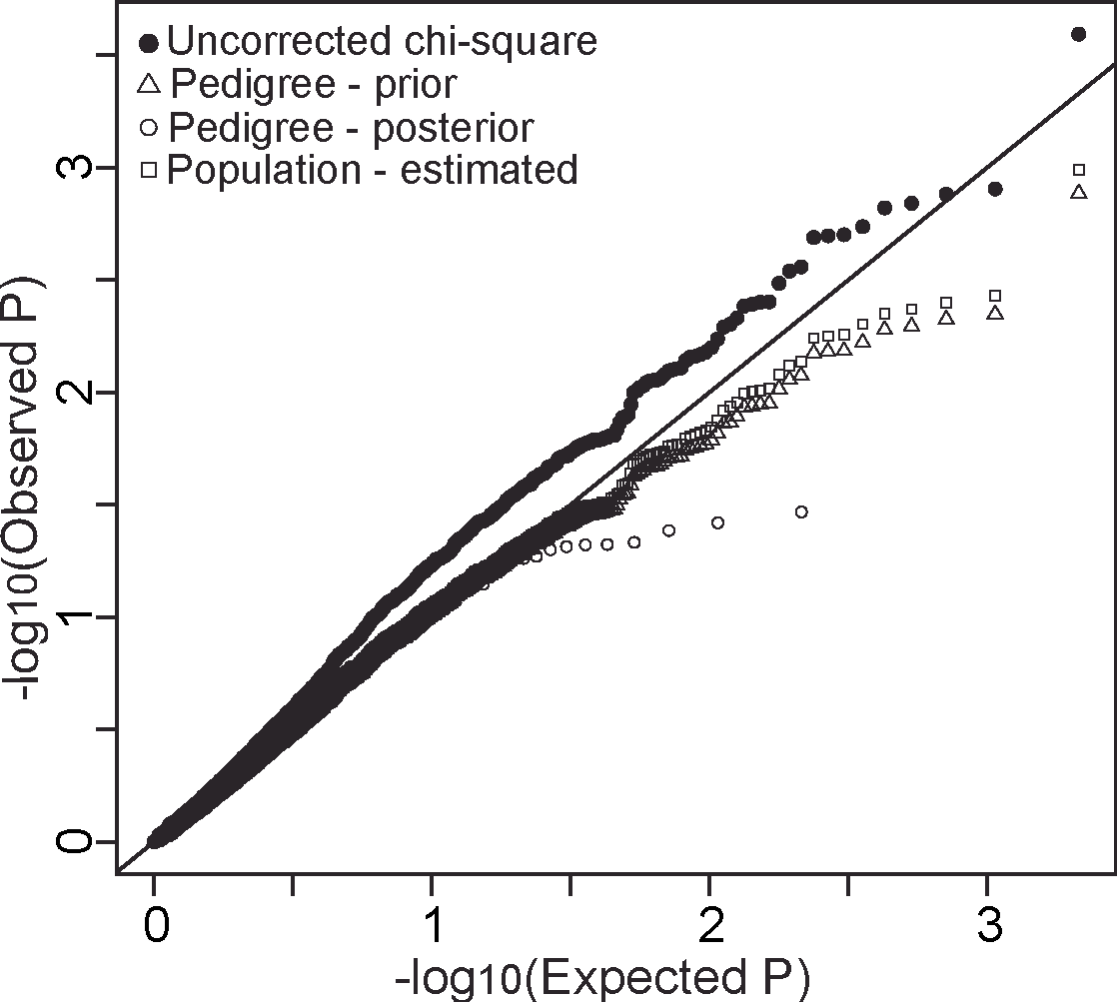
**Case-control significance values with differing corrections for relatedness**. Quartile-quartile plot showing distribution of *p*-values in our case-control study of association between HDL levels and chromosome 7.

Figure 2 used a quartile-quartile plot to illustrate the effects of relatedness, whether known or cryptic, on case control study design. The naïve, uncorrected chi-square test gave a strong inflation of the test statistic, corresponding to a high false-positive rate. In contrast, correction using either the pedigree- or population-based estimated kinship coefficients led to an effective correction of the test statistic distribution. These two distributions are virtually identical. The pedigree-posterior correction over-corrected the test statistic.

### Linkage analyses

**Figure 3:**
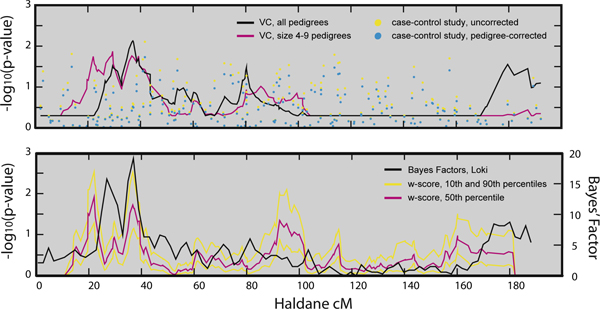
**Summary of HDL linkage and association analyses on chromosome 7**. A, VC analysis using all pedigrees (black line) or size 4-9 pedigrees (pink line) and case-control results using no correction (yellow dots) or pedigree-prior correction (blue dots). B, 50^th ^percentile (pink line), 10^th ^and 90^th ^percentile (yellow lines) of conditional *w*-score analysis on size 4-9 pedigrees, and Bayes' factors from a single MCMC-based oligogenic linkage analysis using all pedigrees (black line).

Linkage analysis results are summarized in Figure 3. Parametric linkage analyses with lm_multiple revealed no signs of linkage, and are therefore not presented. While no clear signal emerged from the case-control study, all linkage analyses show modest evidence of linkage for HDL on chromosome 7 near 40 cM. This signal was suggestive based on trait resimulation (VC LOD = 1.29 at ~38 cM, *p*-value = 0.0087). All linkage analyses detected one or more peaks in the region between 20 and 40 cM. A single Loki run is represented in Figure 3 as all three runs gave strikingly similar results. Additional peaks near 95 cM and 180 cM were detected by some, but not all, linkage analyses.

Randomized *p*-values summarize test significance as well as uncertainty of the test results. For example, although we find significant evidence for linkage near 38 cM using VC analysis and trait resimulation, the conditional test *p*-values in the same region are uncertain, as indicated by the range of *p*-values estimated at that position. To resolve these uncertainties, we would need to use markers at greater density or with greater polymorphism levels to infer the inheritance vectors in this region.

## Discussion

As a rule, more marker data increases the stability and apparent accuracy of IBD estimates (Figure 1), as the number of pairs of independent people with Φ > 0.2 declined dramatically with increasing numbers of loci analyzed. The distribution of loci across the genome also influenced the estimated IBD sharing between pairs of independent people, as 214 markers from across the genome identified fewer pairs of independent people with *k*_1 _> 0.8 than 214 markers from chromosome 7.

However, a relatively modest number of markers were needed to achieve this stability. Although we see a dramatic difference between the *k*-statistics estimated from 214 vs. 1000 genome-wide markers, little difference is observed between estimates from 1000 vs. 3465 genome-wide markers. This suggests that we can thin our marker panels to avoid the effects linkage disequilibrium without strongly altering our inferred *k*-statistics. This also allows for the generation of multiple, equivalent, thinned data sets from a single dense data set, to be used for replication purposes.

Even with thousands of markers, relationships between some pairs of independent people were detected by several approaches. Some of these pairs shared pedigree numbers but could not be connected in the pedigree file. This suggests that our methods were able to detect real relatives even when they were not labelled as such. However, some pairs had unique pedigree numbers, suggesting some cryptic relatedness among the FHS participants and raising the possibility that adjustment for such relationships might be necessary in some analyses.

Relatedness, known or cryptic, clearly inflated uncorrected *p*-values in the case-control study. Fortunately, our corrections using pedigree- and marker-estimated relatedness worked well. Because many case-control studies do not have access to pedigree data, this suggests that our method may be applied to genome-wide association studies without the need for additional pedigree information. The pedigree-posterior estimate of relatedness overcorrected our test statistic, although as the analysis used only the thinned chromosome 7 markers, this is not surprising given the results in Figure 1.

The number and strength of linkage signals varied across methods and by the amount of data used. Linkage analyses, but not the case-control analyses, provided evidence for HDL loci on chromosome 7. The bimodality of the linkage signal between 20 and 40 cM was more clearly defined with the more computationally intensive and trait-model-based *w*-score and MCMC-based oligogenic linkage analyses. The *w*-score also identified a possible linkage signal near 95 cM, although the confidence in actual *p*-values varied across the chromosome. Analysis of the size 4-9 pedigrees emphasized the peak near 20 cM at the expense of the peak near 40 cM, while analyses of all pedigrees identified a modest signal near 180 cM. This signal was detected in a previous GAW [5], suggesting that with more data there may be additional evidence for this locus.

Our novel methods were useful in a variety of situations. Inheritance vectors generated by gl_auto were used in the VC analyses and in empirical significance testing. Analysis of IBD segments identified wide swathes of shared chromosomal regions between pairs of independent people with patterns not visible in a single summary statistic. The method to correct case-control studies for relatedness was practical and effective, and the *w*-score provided both localization and confidence of information in linkage analysis. Although encompassing a wide range of approaches, these methods show clear promise for future work.

## Conclusion

The use of additional data and analytical methods of increasing complexity appears to have paid dividends. However, there are clearly limits. More markers provide better IBD sharing estimates, but a marker density greater than between 1 and 3 cM would likely give only a slight improvement. Correcting case-control studies for relatedness is effective, relatively simple, and can be done using marker data alone. Linkage analyses of greater complexity identified more, albeit weak, linkage signals than simpler analyses. It would appear that all association and linkage methods are capable of detecting strong and clear signals. Because not all studies are fortunate enough to have strong signals, sophisticated analytical tools and large, but not too large, data sets may deliver additional results along with their complexity.

## List of abbreviations used

BMI: Body mass index; FHS: Framingham Heart Study; GAW: Genetic Analysis; Workshop; HDL: High-density lipoprotein; IBD: Identity by decent; MCMC: Markov-chain Monte Carlo; QTL: Quantitative trait locus; SNP: Single-nucleotide polymorphism; VC: Variance components.

## Competing interests

The authors declare that they have no competing interests.

## Authors' contributions

All authors participated in study design and approved the final manuscript. EAT, FB, and YC participated in the IBD analyses, and YC performed the case-control study. EEM, YD, CC, MS, and EMW participated in the linkage analyses. EEM drafted the manuscript.
